# Prognostic value of the ABCD^2 ^score beyond short-term follow-up after transient ischemic attack (TIA) - a cohort study

**DOI:** 10.1186/1471-2377-10-50

**Published:** 2010-06-21

**Authors:** Katrin Holzer, Regina Feurer, Suwad Sadikovic, Lorena Esposito, Angelina Bockelbrink, Dirk Sander, Bernhard Hemmer, Holger Poppert

**Affiliations:** 1 Department of Neurology, Klinikum rechts der Isar, Technische Universität, Munich, Germany; 2 Institute for Social Medicine, Epidemiology, and Health Economics, Charité University Medical Centre, Berlin, Germany; 3 Department of Neurology, Medical Park Hospital, Bischofswiesen, Germany

## Abstract

**Background:**

Transient ischemic attack (TIA) patients are at a high vascular risk. Recently the ABCD^2 ^score was validated for evaluating short-term stroke risk after TIA. We assessed the value of this score to predict the vascular outcome after TIA during medium- to long-term follow-up.

**Methods:**

The ABCD^2 ^score of 176 TIA patients consecutively admitted to the Stroke Unit was retrospectively calculated and stratified into three categories. TIA was defined as an acute transient focal neurological deficit caused by vascular disease and being completely reversible within 24 hours. All patients had to undergo cerebral MRI within 5 days after onset of symptoms as well as extracranial and transcranial Doppler and duplex ultrasonography. At a median follow-up of 27 months, new vascular events were recorded. Multivariate Cox regression adjusted for EDC findings and heart failure was performed for the combined endpoint of cerebral ischemic events, cardiac ischemic events and death of vascular or unknown cause.

**Results:**

Fifty-five patients (32.0%) had an ABCD^2 ^score ≤ 3, 80 patients (46.5%) had an ABCD2 score of 4-5 points and 37 patients (21.5%) had an ABCD^2 ^score of 6-7 points. Follow-up data were available in 173 (98.3%) patients. Twenty-two patients (13.8%) experienced an ischemic stroke or TIA; 5 (3.0%) a myocardial infarction or acute coronary syndrome; 10 (5.7%) died of vascular or unknown cause; and 5 (3.0%) patients underwent arterial revascularization. An ABCD^2 ^score > 3 was significantly associated with the combined endpoint of cerebral or cardiovascular ischemic events, and death of vascular or unknown cause (hazard ratio (HR) 4.01, 95% confidence interval (CI) 1.21 to 13.27). After adjustment for extracranial ultrasonographic findings and heart failure, there was still a strong trend (HR 3.13, 95% CI 0.94 to 10.49). Whereas new cardiovascular ischemic events occurred in 9 (8.3%) patients with an ABCD^2 ^score > 3, this happened in none of the 53 patients with a score ≤ 3.

**Conclusions:**

An ABCD^2 ^score > 3 is associated with an increased general risk for vascular events in the medium- to long-term follow-up after TIA.

## Background

After a transient ischemic attack (TIA), patients are at high risk of further vascular events. Whereas recurrence of cerebral ischemia dominates the short-term prognosis after TIA, with the 90-day stroke risk ranging from 4% to 20%,[1-6] cardiovascular disease becomes the major cause of death on long-term follow-up after TIA and ischemic stroke[7]. This observation is consistent with a high prevalence of asymptomatic coronary artery disease (CAD) in patients with TIA and mild ischemic stroke, which has been shown to vary between 28% and 41% in several studies[8-10].

Recently a new scoring system for evaluating the short-term stroke risk after TIA based on five clinical factors (age, blood pressure, clinical features consisting of unilateral weakness or speech impairment, duration of symptoms, diabetes) has been validated and termed the ABCD^2 ^score[11]. Higher scores were significantly associated with an increased stroke risk at 2, 7, and 90 days, and patients accordingly stratified as high (score 6-7), moderate (score 4-5), and low risk (score 0-3). Three subsequent studies have previously validated the predictive value of the ABDC score in identifying TIA patients with a high risk of early stroke and have given proof of its simple applicability in clinical assessment[12-14].

In this study, we aimed to assess the value of the ABCD^2 ^score in predicting both the cerebrovascular and cardiovascular prognosis during medium- to long-term follow-up after TIA.

## Methods

### Patient selection

We identified 262 patients with possible cerebral TIA who had been consecutively admitted to the Stroke Unit of the Department of Neurology between May 2000 and July 2004. For admission to the Stroke Unit patients have to present with a sudden onset of one or more of the following symptoms being suspicious for a cerebrovascular event: hemiparesis, speech disorder, hemianopsia, gait disturbance, vertigo, dysphagia, disturbance of consciousness, deviation of head and/or ocular bulbs. The major part of patients is assigned by the headquarters of the accident ambulance which is skilled in recognizing symptoms of stroke. Only a small proportion of patients is assigned by registered practitioners or seeks medical advice of its own volition in the accident and emergency department of our hospital. The Stroke Unit also takes admission from nursing home facilities. Located in the centre of a German city it serves an urban area.

Diagnosis was made by the attending neurologist before patient selection. TIA was defined as an acute transient focal neurological deficit caused by vascular disease, which completely reversed within 24 hours[15]. Amaurosis fugax was not considered as TIA. To be eligible, patients had to undergo cerebral magnetic resonance imaging (MRI) including diffusion-weighted imaging (DWI) sequences within 5 days after onset of symptoms, which was the case in 225 patients. 49 patients were excluded for the following reasons: competing differential diagnosis as assessed by the attending neurologist, 41 cases (migraine, 8 cases; epilepsy, 7 cases; functional disorder, 5 cases; peripheral dizziness, 4 cases; syncope, 4 cases; hypertensive crisis, 4 patients; others, 9 cases); malignancy requiring active treatment, 7 cases; concomitant participation in a pharmaceutical trial, 1 case. Informed written or oral consent was obtained from all patients at date of follow-up. The study was approved by the local institutional review board ("Ethikkommission der Fakultät für Medizin der Technischen Universität München"). All research carried out in participating subjects was in compliance with the Helsinki Declaration.

### Baseline clinical variables

The ABCD^2 ^score at time of admission was retrospectively calculated by evaluating medical records as follows: age (≥60 years, 1 point); blood pressure on first assessment after TIA (systolic blood pressure (SBP) ≥140 mmHg or diastolic blood pressure (DBP) ≥90 mmHg, 1 point); clinical features of TIA (unilateral weakness, 2 points; speech impairment without weakness, 1 point); duration of symptoms (≥60 minutes, 2 points; 10-59 minutes, 1 point); diabetes (1 point). In accordance with Johnston et al., the ABCD^2 ^score was stratified into three categories (≤ 3 points, low; 4-5 points, moderate; 6-7 points, high)[11].

In addition, the following data were collected: sex; presence of conventional vascular risk factors; and medical history of coronary artery disease (CAD), heart failure, and symptomatic peripheral artery disease (PAD). Hypertension was defined as systolic blood pressure ≥140 mmHg, diastolic blood pressure ≥90 mmHg, or current use of antihypertensive medication; diabetes mellitus as fasting blood glucose ≥126 mg/dL or current use of antidiabetic agents; hypercholesterolemia as total cholesterol ≥240 mg/dL or current use of lipid-lowering medication; nicotine abuse as current or former regular smoking; and atrial fibrillation as history of electrocardiographically documented intermittent or persistent atrial fibrillation. Diagnostic criteria for myocardial infarction (MI) were typical rise and gradual fall (Troponin T) or more rapid rise and fall (CK-MB) of biochemical markers of myocardial necrosis with at least one of the following: ischemic symptoms (e.g. chest pain), development of pathological Q waves on ECG, ECG changes indicative of ischemia (ST segment elevation or depression) or coronary artery intervention (e.g. coronary angioplasty). Acute coronary syndrome (ACS) was defined as acute myocardial ischemic state also encompassing unstable angina and non-ST segment elevation myocardial infarction without measurable changes of biochemical markers of myocardial necrosis. For coding MI and ACS medical reports from other hospitals or family doctors were obtained.

### Ultrasonography protocol

Extracranial Doppler and duplex ultrasonography (ECD) and transcranial Doppler and duplex ultrasonography (TCD) were performed using multi-range Doppler (DWL Multi-Dop; Compumedics Germany GmbH) and duplex ultrasound devices (Siemens Sonoline Elegra; Siemens AG).

ECD findings were classified as stenotic or occlusive if ECD showed at least one stenosis ≥50% or an occlusion of the cervical internal carotid (cICA) or vertebrobasilar (VBA) arteries. TCD findings were classified as abnormal if TCD revealed at least one intracranial stenosis or an occlusion of the distal internal carotid (dICA), middle cerebral (MCA), or posterior cerebral (PCA) arteries, or detected collateral blood flow through the circle of Willis secondary to extracranial lesions. TCD diagnosis of intracranial stenosis was defined by increased peak flow velocities (≥155 cm/s for dICA and MCA; ≥100 cm/s for PCA) with side-to-side differences > 20% and disturbed flow patterns[16].

### MRI protocol

Cerebral MRI was performed within a maximum of 5 days after onset of symptoms in all patients. All MRI scans were obtained using a 1.5-Tesla scanner (Magnetom Symphony; Siemens AG). The imaging protocol included axial T1-weighted, T2-weighted and DWI sequences and in doubtful cases additionally a sagittal or coronal DWI sequence. Apparent diffusion coefficient (ADC) maps were constructed by linear least-squares fit on a pixel-by-pixel basis after averaging the direction-dependent DWI values. DWI scans were considered positive for ischemia if both a hyperintensity on the isotropic b = 1000 scan and a corresponding hypointensity on the ADC map were detectable.

### Clinical endpoints

At a median follow-up of 27 months (minimum 4 months, maximum 64 months, interquartile range [IQR] 18-41 months), all 176 patients were contacted by telephone or mail for evaluation of new vascular events. The data set was completed by information obtained from relatives, attending physicians and/or hospitals. Our main points of interest were cerebral ischemic events (ischemic stroke or TIA), cardiovascular ischemic events (myocardial infarction (MI) or acute coronary syndrome (ACS), surgical or endovascular revascularization procedures in CAD or PAD), and death of vascular or unknown cause. Other vascular events and death of nonvascular cause also were documented. The interviewer was blinded to the ABCD^2 ^score.

### Statistical analysis

All analyses were performed using the SPSS statistical package version 15.0. For statistical analysis, the ABCD^2 ^score was trichotomized into three categories (≤ 3 points, low; 4-5 points, moderate; 6-7 points, high). For interpretation and summary of results, the ABCD^2 ^score was dichotomized into low values (≤ 3 points) versus moderate or high values (> 3 points), as the proportion of patients with high ABCD^2 ^scores of 6 or more points was relatively small. Association of risk factors was assessed by Student *t *test for normally distributed data and χ^2 ^test for categorized variables. Univariate Cox proportional hazards regression model was used to identify variables associated with the occurrence of endpoints. For the combined endpoint of cerebral ischemic events, cardiac ischemic events, and death of vascular or unknown cause, multivariate Cox regression analysis adjusted for ECD findings and heart failure at baseline was performed in addition. As ECD findings were strongly correlated with TCD results and PAD at baseline, no further variables were added to the multivariate analysis. Associations are presented as hazard ratios (HR) with corresponding 95% confidence intervals (CI); *P *< 0.05 was considered as significant. Percentage values are relative to the patient subset with complete data record.

## Results

A total of 176 Caucasian TIA patients were included in the study. Baseline characteristics of the study population are given in Tables 1 and 2. Notably, patients with a moderate or high ABCD^2 ^score were significantly more likely to show DWI signal intensity changes suggestive of cerebral ischemia than patients with a low ABCD^2 ^score. Medical history revealed former ischemic stroke, TIA, or amaurosis fugax in 40 (23.1%) patients. Nine (5.1%) patients experienced a TIA in the month before admission. Distribution of ABCD^2 ^categories was as follows: 0-3 points, 55 (32.0%) patients; 4-5 points, 80 (46.5%) patients; 6-7 points, 37 (21.5%) patients. In 4 patients the ABCD^2 ^score could not be assigned to any category because of missing data on blood pressure and/or diabetes.

**Table 1:** Single Items of ABCD^2 ^Score in study population (n = 172)

Age (years)*	63.3 ± 14.9
≥60 years	113 (62.2%)
S**B**P ≥140 mmHg/ DBP ≥90 mmHg	95 (54.0%)
**C**linical features	
Unilateral weakness	34 (19.3%)
Speech impairment only	97 (55.1%)
**D**uration of symptoms	
10-59 minutes	36 (20.5%)
≥60 minutes	108 (61.4%)
**D**iabetes (*n*)	30 (17.0%)

**Table 2:** Patient characteristics of study population

	ABCD^2 ^≤ 3 n = 55	3 > ABCD^2 ^< 6 n = 80	ABCD^2 ^≥6 n = 37
Sex, female n (%)	26 (47,3)	26 (32,5)	14 (37,8)
Hypertension n (%)	34 (61,8)	57 (71,3)	32 (86,5)
Hypercholesterolemia n (%)	24 (43,6)	40 (50,0)	18 (48,6)
Body mass index mean ± SD	25,8 ± 3,9	25,8 ± 3,8	26,0 ± 4,0
Nicotine abuse n (%)	22(40,0)	41(51,3)	15(40,5)
Atrial fibrillation n (%)	4(7,3)	12(15,0)	7(18,9)
Coronary artery disease n (%)	9(16,4)	16(20,0)	10(27,0)
Heart failure n (%)	4(7,3)	4(5,0)	3(8,1)
Peripheral artery disease n (%)	3(5,5)	7(8,8)	3(8,1)
DWI abnormality n (%)	9(16,4)	23(28,8)	16(43,2)
ECD: stenotic/occlusive n (%)	8(14,5)	15(18,8)	10(27,0)
TCD: abnormal n (%)	4(7,3)	11(13,8)	5(13,5)

*Mean ± standard deviation.

SBP: systolic blood pressure. DBP: diastolic blood pressure. ECD: extracranial Doppler and duplex ultrasonography. TCD: transcranial Doppler and duplex ultrasonography.

DWI showed signal intensity changes suggestive of cerebral ischemia in 49 (28.3%) patients. ECD detected stenoses ≥50% or occlusions of the cICA or VBA in 34 (19.3%) patients. Six (3.4%) patients had a high-grade cICA stenosis as defined by a local degree of stenosis ≥80%. Five of these six patients subsequently underwent carotid endarterectomy and 1 underwent stent-supported angioplasty. TCD revealed intracranial stenoses in 14 (8.6%) patients and reactive collateral blood flow due to cICA stenosis in 6 (3.7%) patients. In 13 (7.4%) patients, TCD could not be applied because of inadequate temporal bone windows.

Follow-up data were available for 173 (98.3%) patients. In 9 (5.7%) patients an ischemic stroke and in 14 (8.8%) a new TIA was diagnosed. 9 (5.7%) more patients reported symptoms consistent with cerebral ischemia but did not seek medical aid or had competing differential diagnoses as reported by attending physicians and/or hospitals. No patient experienced a new cerebral ischemic event before study MRI. Three (1.8%) patients were diagnosed with acute MI and 2 (1.2%) with ACS; a further 4 (2.4%) patients underwent surgical or endovascular revascularization in CAD, and 1 (0.6%) patient had bypass surgery in PAD. Additionally, four (2.4%) patients suffered from their first-ever angina pectoris attack, and 10 (6.0%) patients experienced other non-ischemic vascular events (cardiac syncope, 4 cases; pacemaker implantation, 2 cases; aortic valve surgery, 1 case; Wolff-Parkinson-White syndrome, 1 case; deep vein thrombosis, 1 case; pulmonary embolism, 1 case). At the time of follow-up, 15 (8.5%) patients had died for the following reasons: cardiac failure, 3 (1.7%); malignancy, 3 (1.7%); pneumonia, 2 (1.1%); unknown cause, 7 (4.0%). No cardiovascular ischemic event happened within the first 90 days after index TIA.

Results of univariate Cox regression analysis are shown in Table 3. Notably, moderate or high ABCD^2 ^scores were significantly associated with the combined endpoint of cerebral ischemic events, cardiac ischemic events, and death of vascular or unknown cause (hazard ratio (HR) 4.01, 95% confidence interval (CI) 1.21 to 13.27, *P *= 0.02). After adjustment for ECD findings and heart failure there remained a strong trend, but this did not reach significance (HR 3.13, 95% CI 0.94 to 10.49, *P *= 0.06). Of the single ABCD^2 ^factors, only the presence of unilateral weakness was significantly associated with the combined endpoint in univariate analysis (HR 3.37, 95% CI 1.00 to 11.30, *P *= 0.049), but there was also a trend for patients aged ≥60 years (HR 2.15, 95% CI 0.88 to 5.26, *P *= 0.09).

**Table 3:** Cox regression analysis of individual risk factors for new vascular events

	Cerebral ischemic event		Cardiovascular ischemic event		Cerebral/cardiac ischemic events, or death of vascular/unknown cause	
	
	HR	95% CI	HR	95% CI	HR	95% CI
3 < ABCD^2 ^> 6	2.71	0.76-9.60	-*	-*	**3.91**	**1.14-13.34**
ABCD^2 ^≥6	2,61	0.62-10.93	-*	-*	**4.26**	**1.13-16.08**
Age ≥60 years	1.30	0.50-3.35	5.30	0.67-41.89	2.15	0.88-5.26
SBP ≥140 mmHg/DBP ≥90 mmHg	0.88	0.35-2.18	-*	-*	1.28	0.57-2.84
Unilateral weakness	4.14	0.96-17.92	1.80	0.20-16.10	**3.37**	**1.00-11.30**
Speech impairment only	1.39	0.20-9.90	8.68	0.96-78.42	2.62	0.66-10.47
Duration 10-59 min	1.32	0.37-4.70	1.83	0.30-10.97	1.63	0.53-4.99
Duration ≥60 min	0.80	0.25-2.50	0.64	0.12-3.48	1.00	0.37-2.72
Diabetes	1.46	0.49-4.33	**4.94**	**1.41-17.30**	1.83	0,79-4.26

Sex, female	1.13	0.49-2.62	0.93	0.23-3.76	1.18	0.56-2.45
Hypertension	0.99	0.39-2.51	-**	-**	1.20	0.54-2.68
Hypercholesterolemia	0.57	0.24-1.34	2.51	0.65-9.70	0.51	0.24-1.10
Body mass index	0.30	0.04-2.24	1.06	0.21-5.26	0.52	0.15-1.76
Nicotine abuse	1.30	0.57-2.94	0.99	0.27-3.69	1.06	0.53-2.12
Atrial fibrillation	0.94	0.28-3.17	-**	-**	1.02	0.35-2.93
Coronary artery disease	0.59	0.18-1.99	2.27	0.62-8.24	0.96	0.41-2.24
Heart failure	3.39	1.00-11.55	2.77	0.34-22.63	**3.97**	**1.51-10.45**
Peripheral artery disease	**7.64**	**2.96-19.71**	-**	-**	**7.42**	**3.25-16.94**
DWI abnormality	1.23	0.51-2.93	0.59	0.12-2.85	0.84	0.37-1.90
ECD: stenotic/occlusive	**4.39**	**1.93-9.99**	**3.73**	**1.05-13.31**	**4.18**	**2.04-8.59**
TCD: abnormal	**4.99**	**1.97-12.62**	**9.62**	**2.46-37.68**	**5.13**	**2.26-11.67**

* Statistical analysis not possible owing to absence of events in one group.

** Statistical analysis not possible owing to small patient numbers.

SBP: systolic blood pressure. DBP: diastolic blood pressure. ECD: extracranial Doppler and duplex ultrasonography. TCD: transcranial Doppler and duplex ultrasonography.

As no cardiovascular ischemic events happened in patients with an ABCD^2 ^score ≤ 3 or an initial blood pressure < 140/90 mmHg, the association between moderate or high ABCD^2 ^scores or hypertensive blood pressure values and the occurrence of cardiovascular ischemic events could not be assessed by statistical analysis. Of the other single ABCD^2 ^factors, only diabetes was significantly associated with new cardiovascular ischemic events in univariate analysis (HR 4.94, 95% CI 1.41 to 17.30, *P *= 0.01). We also observed a trend in patients aged ≥60 years (HR 5.30, 95% CI 0.67 to 41.89, *P *= 0.11) and for patients who developed speech impairment without weakness (HR 8.68, 95% CI 0.96 to 78.42, *P *= 0.05).

The presence of moderate or high ABCD^2 ^scores (HR 2.73, 95% CI 0.81 to 9.29, *P *= 0.11) or unilateral weakness (HR 4.14, 95% CI 0.96 to 17.92, *P *= 0.06) tended to be associated with further cerebrovascular ischemic events in univariate analysis, but significance was not reached in either case. There were also no significant associations between any of the other single ABCD^2 ^factors and the occurrence of cerebrovascular ischemic events. Table 4 shows the graded risk of new vascular events related to person-years based on the trichotomized ABCD^2 ^score, with higher rates of both cerebral and cardiovascular ischemic events in patients with moderate and high ABCD^2 ^scores.

**Table 4:** Risk of new vascular events based on the ABCD^2 ^score

	ABCD^2 ^≤ 3 n	3 > ABCD^2 ^< 6 n	ABCD^2 ^≥6 n
Cerebral ischemic event	3 (2.8/100PJ)	12 (7.9/100PJ)	5 (7.4/100PJ)
Cardiovascular ischemic event	0	6 (4.0/100PJ)	3 (4.4/100PJ)
Cerebral/cardiac ischemic events, or death of vascular/unknown cause	3 (2.8/100PJ)	17 (11.2/100PJ)	9 (13.2/100PJ)

PJ: person years

## Discussion

The results of the present study indicate that the ABCD^2 ^score not only predicts short-term stroke risk after TIA[11,17-19] but may also predict the general vascular risk and particularly cardiovascular risk during medium- to long-term follow-up after TIA. To the best of our knowledge, this is the first study evaluating the prognostic value of the ABCD^2 ^score beyond 90 days follow-up after TIA. However, further studies with larger patient cohorts are necessary to confirm this association.

In addition, the present study implies a particularly increased cardiovascular risk in TIA patients with moderate or high ABCD^2 ^scores. Although Cox regression analysis was not possible because of the absence of events in the patient group with low ABCD^2 ^scores, the study data suggest an association between moderate or high ABCD^2 ^scores and the occurrence of new cardiovascular ischemic events on medium- to long-term follow-up after TIA. This is of special importance as cardiovascular disease becomes the major cause of death on long-term follow-up after TIA,[7] and asymptomatic CAD is known to be prevalent in as many as 28-41% of patients with cerebrovascular disease[8-10]. Healthcare professionals are currently encouraged to optimize coronary risk evaluation in patients with TIA and ischemic stroke based on the Framingham Score and the prevalence of carotid artery disease[20]. In clinical practice, however, coronary risk in TIA patients often is not assessed owing to limited time resources. As the ABCD^2 ^score can easily be derived within seconds, and is often calculated in acute TIA patients anyway, further studies with larger patient cohorts should be conducted to assess its value in predicting cardiovascular events after TIA.

Regarding cerebral ischemic events, we found only a non-significant trend toward higher risk in patients with moderate or high ABCD^2 ^scores (HR 2.73, 95% CI 0.81 to 9.29, *P *= 0.11). However, the incidence of subsequent stroke observed in this study (5.7% at a median follow-up of 27 months) was substantially lower than in other recent reports (7-21% at 12 months)[3,6,21,22]. Given that the first 48 hours after TIA are the period of highest stroke risk,[6,11,23] and urgent TIA treatment is associated with an 80% to 90% reduction in early stroke incidence,[24,25] the reduced stroke incidence in our study might be attributed to the optimized TIA patient management in our setting. All recruited patients were admitted to the Stroke Unit and systematically underwent emergency diagnostic procedures and received secondary prevention therapies. This medical approach is routine practice in our academic center, but differs from standard TIA patient care in hospitals without Stroke Units.

The detected association between higher ABCD^2^-scores and increasing risk of cerebral ischemic events could be attributable to the fact that some aspects of the ABCD score (e.g. unilateral weakness, speech impairment and TIA with prolonged duration) improve the diagnosis of TIA from non-TIA disorders (e.g. syncope or migraine). The remaining features are important vascular risk factors (increasing age, elevated blood pressure and Diabetes) and are therefore likely to be relevant for the cause of future stroke.

Interestingly, TIA patients with moderate or high ABCD^2 ^scores showed an acute ischemic lesion on DWI significantly more often than those with low ABCD^2 ^scores (33.9% vs. 16.7%, *P *= 0.02). Despite emerging evidence of an increased short-term stroke risk in DWI-positive TIA patients,[1,17] we found no association between the detection of an acute ischemic lesion on DWI and the occurrence of cerebral ischemic events during medium- to long-term follow-up. In analogy to the above discussion, both the longer follow-up interval and the high-standard routine management of TIA patients may have weakened the prognostic value of DWI in this study.

There was no increase in the frequency of extracranial stenotic or occlusive disease as assessed by ECD and only a non-significant trend for a higher incidence of abnormal TCD findings in patients with an ABCD^2 ^score > 3. Concordantly, Koton et al. also found no relationship between the ABCD^2 ^score and the prevalence of a carotid stenosis ≥50% in TIA patients[18].

The present study has several limitations. First, a larger patient cohort would have been necessary to improve the statistical power of the study. Moreover, the ABCD^2 ^score was calculated retrospectively on the basis of medical records only and follow-up was conducted as telephone or mail interview only. Concerning the detected association between moderate or high ABCD^2 ^score and higher frequency of DWI signal intensity changes and between moderate or high ABCD^2 ^score and hypertension it has to be acknowledged that these two variables are not independent of ABDC^2^.

## Conclusions

In conclusion, patients with moderate or high ABCD^2 ^scores are at increased risk of suffering from further vascular events in the medium- to long-term follow-up after TIA. This study additionally implies a particularly increased cardiovascular risk in these patients.

## Competing interests

The authors declare that they have no competing interests.

## Authors' contributions

KH and RF carried out the data collection and drafted the manuscript. SS participated in its design and data collection. LE participated in the follow-up data collection and has been involved in drafting the manuscript. AB performed the statistical analyses. DS revised the manuscript critically for important intellectual content and helped to draft the manuscript. BH made substantial contributions to conception, revised the manuscript and gave final approval of the version to be published. HP conceived of the study, and participated in its design and coordination and helped to draft the manuscript. All authors read and approved the final manuscript.

## Pre-publication history

The pre-publication history for this paper can be accessed here:

http://www.biomedcentral.com/1471-2377/10/50/prepub
